# New horizons in the pathogenesis, assessment and management of delirium

**DOI:** 10.1093/ageing/aft148

**Published:** 2013-09-25

**Authors:** Alasdair M. J. Maclullich, Atul Anand, Daniel H. J. Davis, Thomas Jackson, Amanda J. Barugh, Roanna J. Hall, Karen J. Ferguson, David J. Meagher, Colm Cunningham

**Affiliations:** 1 Edinburgh Delirium Research Group, Geriatric Medicine Unit, University of Edinburgh, Room S1642, Royal Infirmary of Edinburgh, 51 Little France Crescent, Edinburgh, UK; 2 Centre for Cognitive Ageing and Cognitive Epidemiology, University of Edinburgh, Edinburgh, UK; 3 Department of Public Health and Primary Care, University of Cambridge, UK; 4 School of Immunity and Infection, University of Birmingham, Birmingham, UK; 5 University of Limerick Medical School, Limerick, Ireland; 6 Trinity College Institute of Neuroscience and School of Biochemistry and Immunology,Trinity College Dublin, Dublin, Ireland

**Keywords:** delirium, older people, delirium pathogenesis, delirium assessment, delirium management

## Abstract

Delirium is one of the foremost unmet medical needs in healthcare. It affects one in eight hospitalised patients and is associated with multiple adverse outcomes including increased length of stay, new institutionalisation, and considerable patient distress. Recent studies also show that delirium strongly predicts future new-onset dementia, as well as accelerating existing dementia. The importance of delirium is now increasingly being recognised, with a growing research base, new professional international organisations, increased interest from policymakers, and greater prominence of delirium in educational and audit programmes. Nevertheless, the field faces several complex research and clinical challenges. In this article we focus on selected areas of recent progress and/or uncertainty in delirium research and practice. (i) Pathogenesis: recent studies in animal models using peripheral inflammatory stimuli have begun to suggest mechanisms underlying the delirium syndrome as well as its link with dementia. A growing body of blood and cerebrospinal fluid studies in humans have implicated inflammatory and stress mediators. (ii) Prevention: delirium prevention is effective in the context of research studies, but there are several unresolved issues, including what components should be included, the role of prophylactic drugs, and the overlap with general best care for hospitalised older people. (iii) Assessment: though there are several instruments for delirium screening and assessment, detection rates remain dismal. There are no clear solutions but routine screening embedded into clinical practice, and the development of new rapid screening instruments, offer potential. (iv) Management: studies are difficult given the heterogeneity of delirium and currently expert and comprehensive clinical care remains the main recommendation. Future studies may address the role of drugs for specific elements of delirium. In summary, though facing many challenges, the field continues to make progress, with several promising lines of enquiry and an expanding base of interest among researchers, clinicians and policymakers.

## Introduction

Over a decade ago, the editors of the book ‘Delirium in Old Age’ [1] stated their concerns about waning interest in delirium. They remarked that in 1970, the word ‘delirium’ was referenced in 2.5% (*n* = 203) of all Medline articles mentioning ‘patient’ or ‘subject’. By 2000, this had fallen to 0.2% (*n* = 253). In 2013 this proportion is the same, and so delirium remains massively understudied in relation to its status as a major cause of ill-health. Nevertheless, the last 5 years has seen much novel research in basic and clinical pathogenesis, neuropsychology and delirium as a predictor of long-term cognitive decline. In parallel with the growing research base, delirium is gaining a higher profile among healthcare professionals. Publication of the UK NICE guidelines [2] in 2010 was a key step forward, and crucially the 2012–13 National Audit of Dementia Care in Acute Hospitals highlighted delirium detection and management as an essential part of good dementia care [3]. Funders and policymakers are also increasingly recognising delirium in the form of targeted grant calls and statements urging that delirium be included in educational programmes, audits and external quality inspections. Two flourishing international professional organisations dedicated to delirium research and practice have been established: the European Delirium Association and the American Delirium Society; both host successful annual meetings. Thus the field is clearly expanding on several fronts as awareness of delirium's enormous impact becomes more widely known. Yet despite these advances, large scale improvements to its clinical care are yet to materialise, and delirium remains almost invisible to the general public.

In this article, we focus on delirium in hospitalised older people. Our goal is not to provide a comprehensive overview but rather to highlight selected areas in which there has been recent progress or that show substantial promise for development.

## Pathogenesis

Delirium is a heterogeneous and fluctuating syndrome resulting mostly from peripheral conditions that precipitate acute brain dysfunction. It is now evident that delirium is also associated with brain injury, because delirium is a strong risk factor for new onset dementia [4] as well as acceleration of existing dementia [5]. Thus, there are several overlapping domains of enquiry. These include understanding how peripheral infections elicit brain dysfunction, the dysfunction causing delirium itself and the processes underpinning the association of delirium with long-term cognitive decline. Given the complexity of the brain and its interactions with the periphery, numerous processes are likely involved. Yet in some cases the causation of delirium and associated brain injury is not difficult to understand, at least conceptually. Delirium commonly results from specific, readily definable insults such as hypoxia, metabolic abnormalities, stroke and drug effects. This broad category of precipitants can be termed ‘direct brain insults’ [6]. In many other cases patients with delirium have not had a significant direct brain insult, for example, where the cause of delirium is an apparently mild peripheral infection or injury that has not led to substantial physiological disturbance. In these situations, it can be assumed that the delirium has been initiated by aspects of the body's response to the insult. We have termed this second category of causes of delirium ‘aberrant stress responses’. Here, ‘stress’ broadly refers to the sympathetic nervous system, hypothalamic–pituitary–adrenal axis, inflammatory pathways and other systems activated in response to acute threat. The term ‘aberrant’ is used because the stress response may be exaggerated and manifestly has adverse effects on the brain.

### Human studies

In humans, exogenous administration of peripheral pro-inflammatory cytokines is known to cause cognitive dysfunction, and even delirium, mediated at least partly by altered neurotransmission. Direct evidence linking inflammatory cytokines and cortisol with delirium comes from studies of blood [7, 8] and cerebrospinal fluid (CSF) [9]. Elevated endogenous levels of serum interleukin (IL)-6 and IL-8 have been found in elderly hospital inpatients who develop delirium, even after correcting for infection and baseline cognitive impairment [10], with similar findings in hip fracture patients [11]. Levels of IFN-ɣ (pro-inflammatory), IL-RA and IGF-1 (anti-inflammatory) are altered in delirium [12, 13]. CSF IL-8 is increased [14]. An exaggerated stress response to acute illness, often combined with impaired negative feedback, results in high cortisol. This is commonly seen with ageing and neurodegenerative disease [6]. Elevated cortisol is an established cause of mental status deterioration and one prominent hypothesis of delirium pathogenesis is that delirium is precipitated by pathologically elevated cortisol occurring with acute stress from illness or surgery [6]. Several studies have investigated this proposition possibility, in serum samples [15], and CSF [16].

How direct brain insults and aberrant stress responses lead to the brain dysfunction underlying delirium is not well understood, though the association of certain classes of drugs with delirium has implicated cholinergic, dopaminergic, adrenergic and GABAergic systems. Evidence of increased dopaminergic signalling has been found in the CSF of delirium patients with psychotic features [17]. Further CSF and functional neuroimaging experiments are required to identify abnormalities in neurotransmission occurring during delirium in humans; animal studies will be useful in suggesting lines of enquiry (see below).

The mechanisms underlying the association between delirium and dementia are under-researched in humans. Post-mortem studies have raised the intriguing possibility that the processes linking delirium and dementia may not be mediated by classical dementia pathology [4]; it is likely that inflammatory pathways are important given the evidence from animal studies [18]. Elevated CSF levels of S100B, a marker of CNS damage derived largely from astrocytes, were reported in active delirium in patients with hip fracture compared with those without delirium [19]. This is consistent with similar findings in blood [20]. Clearly, this is a domain of delirium research that is of great interest given the implications for primary and secondary prevention of dementia.

### Animal studies

Animal models offer enormous potential in delirium pathophysiological research. Of all the mental disorders delirium is particularly amenable to animal studies, because it is acute, severe and measurable, and because clinical studies have provided clear leads on precipitants and factors increasing vulnerability. For example, animal studies are able to directly test the hypotheses, frequently suggested in clinical review articles, that peripheral inflammation induces or synergises with central cholinergic dysfunction to induce transient behavioural change resembling delirium. More sophisticated studies involving combinations of aetiological factors are also possible and these could more closely model the complexities seen in clinical practice. Animal studies can also discover new biomarkers, and identify and test candidate drug treatments.

The first explicit attempt to model delirium in rodents administered the muscarinic receptor antagonist atropine intraperitoneally and demonstrated acute cognitive deficits in a blind alley maze and EEG slowing reminiscent of delirium [21]. More recently, investigations of the role of inflammatory processes have involved mimicking severe sepsis using either high doses of lipopolysaccharide (LPS; 5–10 mg/kg), or by performing caecal ligation and puncture leading to polymicrobial sepsis. Acute alpha activity slowing in EEG and decreased glucose uptake has been demonstrated by microPET at 24 h post-LPS [22]. LPS studies have focused on long-term impairment, demonstrating neuronal death, synaptic loss and spatial memory deficits in rats at 12 weeks post-LPS (10 mg/kg). It is of interest, but so far little studied, that GABAergic anaesthetics, frequently used in ICU patients, may increase mortality in active infection-induced critical illness in rodents [23]. Furthermore, subthreshold scopolamine doses can produce working memory dysfunction in rodents when combined with diazepam [24]. IL-1β contributes to cognitive dysfunction during surgery, infection and sepsis [25, 26] and can directly increase GABA receptor expression and GABAergic tone [27]. Thus, interactions among IL-1β, GABA and ACh are of considerable interest for delirium and require detailed further study.

Recent models addressing inflammatory processes combine much lower levels of systemic inflammation superimposed upon prior brain vulnerability, more closely modelling delirium caused by mild insults in older patients. In aged mice [28] or those with pre-existing neurodegenerative pathology [29], low doses of LPS induced acute and reversible cognitive dysfunction. Microglia primed by primary pathology to produce exaggerated IL-1β responses to subsequent inflammatory stimulation [30] were implicated in the acute cognitive deficits in both of these studies. Although the role of microglial priming remains unproven, activated microglia robustly expressed COX-1 and the described cognitive deficits were dependent on both COX-1-mediated prostaglandins and IL-1β [31]. Animals with selective lesions of the basal forebrain cholinergic system have been demonstrated to be susceptible, upon systemic LPS dosing (100 µg/kg), to acute cognitive dysfunction that was reversible upon inflammatory resolution [32]. These deficits were largely prevented by treatment with the acetylcholinesterase inhibitor donepezil [32]. These data suggest a role for acetylcholine on a background of neurodegenerative pathology in the cholinergic system. How systemic inflammatory mediators and brain vulnerability might interact during sickness behaviour syndrome to produce delirium has been more comprehensively reviewed elsewhere [8].

## Prevention

Prevention strategies are typically based on identifying high-risk patients, and addressing modifiable risk factors in these patients. In some settings, such as geriatrics wards, most patients can be considered high risk. In settings with a wider range of levels of vulnerability several delirium risk scales have been validated, aimed at appropriate risk stratification and resource allocation. These include scales developed for the Intensive Care Unit (ICU) (PRE-DELIRIC [33]), cardiothoracic [34] and vascular surgery units [35]. Several overlapping delirium prevention programmes focused on non-pharmacological measures have been tested [2, 36–40] and generally they have been found to be cost-effective [41]. Delirium prevention by the use of specific drugs shows some promise [42] although these studies have been restricted to surgical patients [43]. Currently, pending a stronger body of positive evidence, pharmacological prevention is not advocated in routine clinical practice. Future definitive studies will likely be large and complex, because of the many difficulties involved in study design. For example, in existing randomised controlled trials of pharmacological prevention, the heterogeneity of the clinically managed control arms limits study comparability. Measurement of effect in drug studies also requires care because most delirium scales are biased towards hyperactive symptoms; thus the sedation caused by antipsychotic drugs may improve the overall scale score while leaving any positive or negative effects on hypoactive symptoms inadequately assessed. Conversely, some commentators have noted that the effect of antipsychotics is independent of sedative activity and temporally inconsistent with the antipsychotic effect observed in functional psychoses, thus pointing towards more direct actions in preventing or treating the syndrome of delirium [44].

Future research on delirium prevention should address several issues. It is still unclear if interventions are more efficacious in higher risk patients. Related to this, tailored delirium prevention as advocated by the NICE guidelines [2] requires further detailed evaluation. It is plausible that some highly vulnerable patients could benefit from more resource-intensive tailored prevention packages; stratification of risk would make realisation of such interventions in clinical practice more feasible. More broadly, it is important to clarify if prevention and treatment are meaningfully distinct approaches, because incident and prevalent delirium may overlap in prodromal or subsyndromal forms. Another unresolved problem is how to translate existing research findings into routine care. Studies that consider low cost, practical implementation in mainstream healthcare are still lacking. However, some elements of multicomponent programmes are easier and cheaper to implement than others, and healthcare providers should consider introducing these components into practice now, rather than adopting an all-or-none approach for a particular package of measures. For example, urging staff to provide hearing aids and glasses, involving family members in care, providing frequent orientation, ensuring adequate hydration and nutrition, detecting and treating pain and constipation, avoiding urinary catheterisation, and avoiding deliriogenic drugs, are readily achievable in many settings without additional staffing. Other elements, such as cognitive stimulation, may be more difficult and costly to implement.

## Assessment

Under-detection of delirium is well documented: at least two-thirds of cases are missed [45, 46]. Older age, the presence of comorbid neuropsychiatric disorders, prominent pain and hypoactive presentation contribute to decreased recognition. Diagnosis etection is also hindered by confusing nomenclature, with many terms describing acute brain disturbance in use in different populations and treatment settings. The use of ‘delirium’ as an umbrella term is important in engaging and educating colleagues throughout the healthcare spectrum. A second barrier to delirium assessment is the wide variety of detection tools available. This reflects the clinical heterogeneity of the condition and the varying skills of assessors with identification of some delirium through observed behaviours, and in other cases by detailed cognitive assessment requiring more expertise [47]. While the agitated patient is easily identified, the more common presentation of hypoactive delirium is frequently missed. For this reason, regular mental status assessment needs to become a ‘vital sign’ embedded into basic hospital care. One important advance in the UK is the adoption of a new National Early Warning Score, which incorporates a 4-point level of consciousness measure [48]. Some recent evidence suggests that the reduced level of consciousness is a specific (though not sensitive) sign of delirium [49, 50], and so it could become a stimulus for specific delirium screening, bolstering rates of detection. These and other and system-wide approaches, embedded into routine practice, are likely required to address the huge unmet need for delirium assessment [51].

Whatever triggers are used, further assessment can be made using a validated tool, such as the 4 “A”s Test (4AT; Bellelli *et al*., submitted), the Confusion Assessment Method or its variants [52, 53], the Nursing Delirium Screening Scale [54] or others. Selection of the tool depends on multiple factors, including the time available, the level of tester skill, and the clinical setting. In most general hospital environments, the most successful assessment tools are likely to be simple, quick and usable without extensive training in delirium. Lessons could be learnt from the Netherlands, where the Delirium Observation Screening scale [55] has been implemented in <50% of hospitals. Assessment tools that discriminate between delirium and related neuropsychiatric conditions, especially dementia or depression are important, yet the ability of commonly used screening tools to achieve this is poorly studied [56].

Specialist delirium assessment tools and investigations may be appropriate in a minority of patients with delirium where the precipitant is unclear or the clinical course unusual. The Memorial Delirium Assessment Scale [57], Delirium Rating Scale–Revised–98 [58] and Cognitive Test for Delirium [59] enable detailed assessment to detect delirium in cases involving related neuropsychiatric conditions. Future developments in objective assessment of attention [60], and more detailed assessment of the level of consciousness [49] may assist delirium detection. EEG may show moderate to severe generalised slowing of brain activity in delirium, distinguishing the condition from dementia [61] and may also detect non-convulsive status epilepticus.

Neuroimaging is routinely used in clinical practice to exclude primary neurological causes of delirium such as stroke, haemorrhage and tumours. Yet the evidence base informing decisions on neuroimaging in delirium is very small; a recent review of the literature found only 16 studies with just 350 patients with documented delirium scanned in total [62]. This is surprising given that in some centres 50% or more of patients with delirium undergo CT scanning. This matters not only because of possibly inappropriate use of resources, but also because CT scanning may be stressful for some patients. Future research has a role in clarifying the benefits of CT scanning in clinical practice [63]. Additionally, MRI or other modalities could shed light on predisposing factors, neural substrates of delirium and consequences of delirium. Lumbar puncture currently only has a clinical role in excluding specific CNS causes of delirium. Research may yield useful biomarkers of CNS inflammation or damage that could be used in future clinical practice [14].

## Management

Guidance on the management of delirium is ubiquitous in general medical and geriatrics textbooks as well as in delirium review articles. Yet proving the effectiveness of delirium treatment has been surprisingly difficult, and there is little direct positive evidence available. For example, a recent large RCT showed significant effects on patient and care experience, but not on mortality, length of stay and other outcomes [64]. Because of the lack of evidence, recommendations on management have come from expert consensus. There is broad agreement with respect to most aspects of care [2, 65], including: treating all the probable acute causes; reducing the impact of predisposing factors (such as sensory impairments); optimising physiological conditions for the brain (e.g. providing adequate oxygen delivery and stopping or reducing deliriogenic drugs); treating the syndrome itself, through providing a stable and reassuring environment and sometimes using drugs for agitation and distress (see below); avoiding complications such as aspiration pneumonia and prolonged immobility; providing rehabilitation; and communicating effectively with families. Future research might involve structured programmes of intensive intervention along these lines, though deciding which treatment arm components are not offered to patients randomised to usual care raises complicated ethical and scientific challenges.

The evidence base on pharmacological intervention in delirium is slight. Small randomised controlled trials of quetiapine in medical and ICU patients suggest a positive effect, but more robust studies are required [66, 67]. Conventional management often involves low dose haloperidol, though this reflects tradition rather than the availability of positive studies. There is currently little evidence in favour or against the use of newer antipsychotics, but a study of risperidone in subsyndromal delirium shows some promise [68, 69]. Many practitioners already use risperidone and other newer antipsychotics, however [65]. In current practice, consideration of efficacy (and principal modes of action) versus significant risk of adverse effects in often frail elderly patients, means that antipsychotics are used mainly where non-pharmacological methods have failed to control distressing psychotic or other neuropsychiatric symptoms or the safety of the patient or others is compromised. The optimal duration of such treatment is uncertain, but short-term exposure with treatment discontinuation soon after symptoms settle is preferred [44, 70]. However, evidence of post-delirium recall suggests psychotic phenomena may warrant more aggressive treatment, even among hypoactive patients [71]. This is more controversial, as antipsychotic medications used to treat mild agitation may promote hypoactive delirium. Additional studies are required, perhaps targeted to components of the delirium syndrome, such as psychosis [42, 72].

High quality delirium care is complex and time-consuming, and in all but the mildest and briefest cases requires specialist expertise. For some patients, management may be best delivered in specialist ‘delirium rooms’ providing integrated medical and psychiatric care in an optimised, secured environment. In addition to the theoretical advantages, evidence indicates that such approaches reduce the length of delirium and increase the likelihood of recovery to independent living [73]. Some of these benefits are achieved by specialist geriatric care units, which have a wider remit for function-focused care [74] but given the high prevalence of delirium within hospitals these are only available to a proportion of delirium sufferers. Therefore, although specialist delirium units may have a role in complex cases, ensuring better whole-hospital care is also necessary. Some suggestions regarding the features of a ‘Delirium-Friendly Hospital’ are provided in Box 1.

**Features of a Delirium-Friendly Hospital**

- Staff aware that delirium affects 1 in 8 of their patients
- Staff all have basic knowledge of delirium
- Delirium screening is routine
- Delirium information for patients and families is available
- The hospital has a delirium pathway
- Basic delirium prevention measures are in place
- Environment: orientation information, signage, etc.
- Awareness of value of role of family/carers
- Availability of expert specialist care

The management of delirium should include strong consideration of post-discharge follow-up, given the frequency of persistent delirium at discharge and strong association with future dementia risk [4, 75]. This is a neglected area of delirium care, given that studies have shown how many patients' understanding of the episode can be coloured by feelings of embarrassment or worries that they were ‘senile’ or ‘mad’ [76]. Moreover, post-delirium recall can produce a condition similar to post-traumatic stress disorder, requiring psychological support [71, 77]. Another research and practice priority is to understand why some episodes of delirium are so persistent and who is at a greater risk of this and of post-delirium cognitive decline, either through risk stratification or biomarker development. Early identification of such patient will help to focus treatment and in offering prognostic information for patients and families.

## Future directions

Delirium poses considerable challenges in multiple domains of science and healthcare. Much remains to be discovered. Yet clinical implementation of prevention, detection and treatment strategies is a clear imperative, for the following reasons: (i) research has shown that delirium prevention is effective; (ii) detection of established delirium is essential to relieve distress [78] and improve communication with families; (iii) though specific delirium treatment is not well supported by formal studies, clinical experience strongly suggests that identifying and treating causes and providing comprehensive supportive care is efficacious.

The research priorities are broad [72, 79, 80]. Recent animal model research has suggested possible mechanisms [18], and even treatment strategies. Increased capacity for animal model research is urgently required. Understanding the relationship between delirium and dementia is a primary target for mechanistic research, given the potential for primary or secondary prevention of dementia and with many important questions outstanding, such as the effects of delirium severity, duration, and presumed aetiology as modifiers of the relationship. Although several instruments for delirium assessment are already available in research and practice, further work could focus on the development of objective cognitive tests and more detailed assessments of the level of consciousness. The benefits would include more finely grained measures for clinical trials, and more precise assessment of the effects of treatments on subcomponents of delirium. Positive evidence in favour of delirium treatment programmes would powerfully stimulate efforts to improve routine care; to some extent this is now being observed as a positive consequence of the solid evidence supporting delirium prevention.

## Conclusions

Delirium is one of the major unmet medical needs in modern clinical practice. Understanding of the characteristics, causes and consequences of delirium has grown considerably in recent decades. Advances in knowledge of mechanisms, treatment and implementation of prevention and detection strategies are current priorities. The expanding base of interest among researchers, clinicians and policymakers evident in recent years give rise for cautious optimism that there will be progress on these fronts.

**Key points**

- Delirium is one of the major unmet medical needs in modern clinical practice.
- Delirium strongly predicts future new-onset dementia and accelerates existing dementia.
- Delirium prevention is effective but implementation in clinical practice is still lacking.
- Animal model research has provided valuable insights into possible mechanisms.
- Higher detection rates of delirium in routine practice remains a major priority.

## Conflicts of interest

AMJM has patents pending for computerised tests of attentional impairments in delirium.

## Funding

A.M., D.D., T.J., A.B., R.H. and K.F. are members of the University of Edinburgh Centre for Cognitive Ageing and Cognitive Epidemiology, part of the cross council Lifelong Health and Wellbeing Initiative. Funding from the BBSRC, EPSRC, ESRC and MRC is gratefully acknowledged. D.D. is supported by a Wellcome Trust Clinical Fellowship. T.J. is supported by the Research into Ageing Fund (grant number 367), a fund set up and managed by Age UK and the British Geriatrics Society. A.B. is supported by The Dunhill Medical Trust (grant number RTF17/0111). R.J.H. is supported by Research into Ageing and the British Geriatrics Society (grant number 342). C.C. is supported by a Wellcome Trust Senior Research Fellowship.
